# The gut hormone glucose-dependent insulinotropic polypeptide is downregulated in response to myocardial injury

**DOI:** 10.1186/s12933-022-01454-3

**Published:** 2022-02-05

**Authors:** Florian Kahles, Matthias Rau, Martin Reugels, Ann C. Foldenauer, Robert W. Mertens, Maria C. Arrivas, Jörg Schröder, Paul Idel, Julia Moellmann, Emiel P. C. van der Vorst, Nikolaus Marx, Michael Lehrke

**Affiliations:** 1 Department of Internal Medicine I-Cardiology, University Hospital Aachen, Pauwelsstraße 30, 52074 Aachen, Germany; 2 Department of Medical Statistics, University Hospital Aachen, Pauwelsstraße 19, 52074 Aachen, Germany; 3 Fraunhofer Institute for Molecular Biology and Applied Ecology IME, Theodor-Stern-Kai 7, 60596 Frankfurt am Main, Germany; 4 Interdisciplinary Center for Clinical Research (IZKF), Institute for Molecular Cardiovascular Research (IMCAR), University Hospital Aachen, Pauwelsstraße 30, 52074 Aachen, Germany; 5 Department of Pathology, Cardiovascular Research Institute Maastricht (CARIM), Maastricht University Medical Centre, Universiteitssingel 50, 6229 ER Maastricht, The Netherlands; 6 Institute for Cardiovascular Prevention (IPEK), Ludwig-Maximilians-University Munich, Pettenkoferstraße 9, 80336 Munich, Germany; 7 Department of Internal Medicine, University Hospital Aachen, Pauwelsstraße 30, 52074 Aachen, Germany

**Keywords:** Incretin, GIP, Myocardial infarction, Cardiac surgery, Cardiac injury

## Abstract

**Background:**

The gut incretin hormones GLP-1 (glucagon-like peptide-1) and GIP (glucose-dependent insulinotropic peptide) are secreted by enteroendocrine cells following food intake leading to insulin secretion and glucose lowering. Beyond its metabolic function GIP has been found to exhibit direct cardio- and atheroprotective effects in mice and to be associated with cardiovascular prognosis in patients with myocardial infarction. The aim of this study was to characterize endogenous GIP levels in patients with acute myocardial infarction.

**Methods and results:**

Serum concentrations of GIP were assessed in 731 patients who presented with clinical indication of coronary angiography. Circulating GIP levels were significantly lower in patients with STEMI (ST-elevation myocardial infarction; n=100) compared to clinically stable patients without myocardial infarction (n=631) (216.82 pg/mL [Q1–Q3: 52.37–443.07] vs. 271.54 pg/mL [Q1–Q3: 70.12–542.41], p = 0.0266). To characterize endogenous GIP levels in patients with acute myocardial injury we enrolled 18 patients scheduled for cardiac surgery with cardiopulmonary bypass and requirement of extracorporeal circulation as a reproducible condition of myocardial injury. Blood samples were drawn directly before surgery (baseline), upon arrival at the intensive care unit (ICU), 6 h post arrival to the ICU and at the morning of the first and second postoperative days. Mean circulating GIP concentrations decreased in response to surgery from 45.3 ± 22.6 pg/mL at baseline to a minimum of 31.9 ± 19.8 pg/mL at the first postoperative day (p = 0.0384) and rose again at the second postoperative day (52.1 ± 28.0 pg/mL).

**Conclusions:**

Circulating GIP levels are downregulated in patients with myocardial infarction and following cardiac surgery. These results might suggest nutrition-independent regulation of GIP secretion following myocardial injury in humans.

## Introduction

The gut incretin hormones GLP-1 (glucagon-like peptide-1) and GIP (glucose-dependent insulinotropic peptide) are secreted by enteroendocrine cells following nutrient intake leading to insulin secretion and glucose control [1, 2]. Pharmacological activation of the incretin axis is currently used for the treatment of patients with type 2 diabetes [3]. Beyond their glucoregulatory function GLP-1 receptor agonists exert pleiotropic vascular- and cardioprotective effects in different organ systems [4]. For example, we and others found GLP-1 to reduce and stabilize atherosclerotic lesions in ApoE^−/−^ mice by diminishing vascular inflammation [5, 6]. Importantly, six large clinical trials showed improved cardiovascular outcomes in diabetic patients at high cardiovascular risk after treatment with GLP-1-receptor agonists on top of standard antidiabetic therapy [7–12]. In contrast, the role of the other incretin hormone GIP for cardiovascular disease (CVD) remains largely unknown. Endogenous GIP stimulates glucose-dependent insulin secretion more potently than GLP-1 [13, 14]. This effect is however lost in patients with diabetes [15, 16]. Further, GIP suppresses glucagon secretion in states of hyperglycemia while stimulating glucagon release in hypoglycemic situations [17]. Recent efforts have led to the development of combined GLP-1/GIP receptor co-agonists which in clinical trials were importantly found to have more potent blood glucose lowering and body weight reducing effects than sole GLP-1 receptor agonists [18]. Cardiovascular outcome trials (i.e. SURPASS-CVOT; NCT04255433) investigating whether the superiority of Tirzepatide vs. GLP-1 receptor agonists can be translated into improved cardiovascular prognosis are ongoing. Posthoc-Analyses of recent clinical trials yielded encouraging results by showing that the reduction in cardiovascular risk markers as hsCRP, MCP-1 and ICAM-1 was stronger by dual GIP and GLP-1 receptor agonism vs. sole GLP-1 receptor agonism [19]. Understanding the cardiovascular functionality of GIP therefore seems of great interest. First experimental studies suggested GIP to have beneficial cardiovascular effects in rodents [20–23]. Recently we found circulating GIP levels in patients with acute myocardial infarction to be associated with cardiovascular prognosis. In these patients lower GIP levels independently predicted adverse outcome (cardiovascular death) [24]. These findings might suggest a cardiovascular protective role of the endogenous GIP system. The aim of this study was to characterize endogenous GIP levels in patients with acute myocardial infarction.

## Methods

### Clinical study I

We analyzed blood samples from 731 patients of our cardiovascular biobank (559 male and 172 female), who underwent coronary angiography at the University Hospital Aachen (Department of Cardiology). The only exclusion criterion was failure to give written informed consent. Patients were divided into two groups—patients with acute myocardial infarction (STEMI = ST-elevation myocardial infarction; n = 100) compared to clinically stable patients without myocardial infarction (n = 631). Of all patients with STEMI (n = 100) two patients died within 2 days after enrollment and were classified as patients with fatal acute myocardial infarction. Blood was collected in a random non-fasting manner. After centrifugation at 2000 *g* at 4 °C for 20 min, serum aliquots of 1 mL were frozen immediately at −80 °C. Total GIP serum levels were determined by using a commercial ELISA kit (Millipore) according to the manufacturers’ instructions. Study protocols and biosampling were approved by the local ethics committee (RWTH University Hospital Aachen) and conducted in accordance with the ethical standards laid down in the 1964 Declaration of Helsinki.

## Clinical study II

We enrolled 18 patients (11 male, 7 female) scheduled for cardiac surgery with cardiopulmonary bypass and requirement of extracorporeal circulation as a reproducible condition of myocardial injury at the University Hospital of Munich (Campus Grosshadern). Patients were excluded from the study if they met the following criteria: failure to give written informed consent, pregnancy, diabetes mellitus, fasting glucose >126 mg/dL, use of antidiabetic medication or glucocorticoids.

Blood samples were drawn directly before surgery (baseline), upon arrival at the intensive care unit (ICU), 6 h post arrival to the ICU and at the morning of the first and second postoperative days.

Patients were fasted since the evening of the preoperative day. Glucose levels were assessed on an hourly basis and insulin-infusion rate consequently adjusted to maintain glucose levels between 80 and 126 mg/dL. No glucose containing solutions were given during the day of the procedure, while all patients received continuous infusion of glucose 10% with a rate of 10 ml/h at the morning of the first postoperative day. No additional parenteral or enteral nutrition was administered during the observation period. After centrifugation at 2000*g* at 4 °C for 20 min, serum aliquots of 1 mL were frozen immediately at −80 °C. Total GIP serum levels were determined by using a commercial ELISA kit (Millipore) according to the manufacturers’ instructions. Study protocols and biosampling were approved by the local ethics committee (University Hospital of Munich, Ludwig-Maximilians-University) and conducted in accordance with the ethical standards laid down in the 1964 Declaration of Helsinki.

### Statistical analysis

Continuous data are presented as mean ± SD or SEM and median (Q1-Q3) in case of heavily skewed data. Categorical outcomes are shown as absolute (No.) and relative frequencies (%). A Wilcoxon rank-sum test was performed to analyze the group GIP levels in Clinical study I. In addition, a linear mixed model was computed to adjust for influences of parameters significantly correlated with GIP. The association between GIP and other characteristics was assessed using the Spearman correlation coefficient ρ. To analyze time effects in Clinical study II, a linear mixed model for logarithmized GIP values with time point as an independent factor, a random patient effect and a compound symmetry covariance structure was computed. P-values for the comparison between time points 2–5 to 1 (baseline) were adjusted using the Dunnett–Hsu adjustment for multiple comparisons. The level of significance was set at 5%. Statistical analyses were performed with SAS software version 9.4 (PROC GLIMMIX, PROC NPAR1WAY, PROC MIXED; SAS Institute, Cary NC, USA).

## Results

**Fig. 1:**
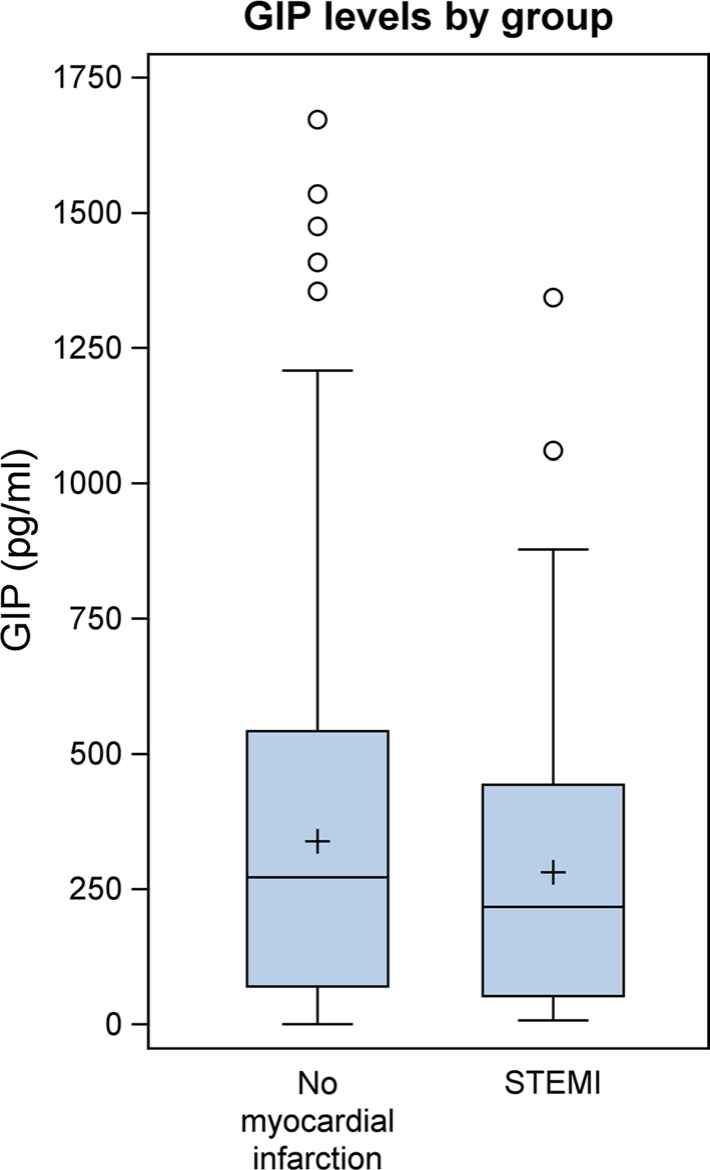
Serum GIP levels are downregulated in patients with acute myocardial infarction (Clinical study I): Circulating serum GIP levels from patients with acute myocardial infarction (STEMI; n = 100) compared to clinically stable patients without myocardial infarction (n = 631). A Wilcoxon rank-sum test was performed (p = 0.0266)

**Fig. 2:**
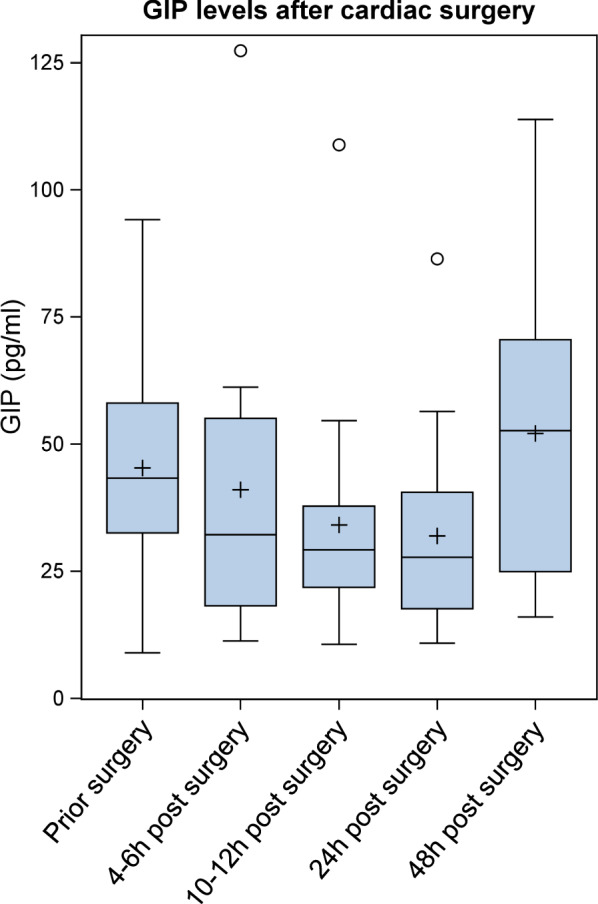
Serum GIP levels are decreased in response to cardiac surgery (Clinical study II): Kinetics of serum GIP levels over time after cardiac surgery (time point 1: at baseline before cardiac surgery, time point 2: at arrival to the ICU (4–6 h post initiation of surgery), time point 3: 6 h post arrival to the ICU (10–12 h post initiation of surgery), time points 4 and 5: in the morning one and two days after surgery). A linear mixed model for logarithmized GIP values with time point as independent factor, a random patient effect and a compound symmetry covariance structure was computed (time point effect p = 0.0105). P-values for the comparison between time points 2– to 1 (baseline) were adjusted using the Dunnett-Hsu adjustment for multiple comparisons (comparison between time point 4 and baseline p = 0.0384)

Clinical and laboratory baseline characteristics and biomarker concentrations are shown in Table 1. The study population comprised 731 patients of our cardiovascular biobank (559 male and 172 female), who underwent coronary angiography at the University Hospital Aachen (Department of Cardiology). Patients were divided into two groups—patients with acute myocardial infarction (STEMI = ST-elevation myocardial infarction; n = 100) compared to clinically stable patients without myocardial infarction (n = 631). GIP levels were not associated with age, sex, kidney function, hypertension, creatine kinase, troponin or WBC (white blood cells) while we observed a significant correlation of GIP levels with BMI (body-mass-index), CRP (C-reactive protein) and type 2 diabetes (Table 2). As shown in Fig. 1; Table 1 circulating GIP levels were significantly lower in patients with STEMI compared to clinically stable patients without myocardial infarction (216.82 pg/mL [Q1–Q3: 52.37–443.07] vs. 271.54 pg/mL [Q1–Q3: 70.12–542.41], p = 0.0266). This association remained significant in a multivariable model after adjustment for all parameters which significantly correlated with GIP levels (BMI, CRP and type 2 diabetes) (p = 0.0311). To further elucidate the underlying mechanisms of downregulated GIP levels during acute myocardial infarction we prospectively enrolled 18 non-diabetic patients (11 male, 7 female) scheduled for cardiac surgery with cardiopulmonary bypass and requirement of extracorporeal circulation as a second reproducible condition of myocardial injury. Blood samples were drawn directly before surgery (baseline), upon arrival at the intensive care unit (ICU), 6 h post arrival to the ICU and at the morning of the first and second postoperative days. Mean circulating GIP concentrations decreased in response to surgery from 45.3 ± 22.6 pg/mL at baseline to a minimum of 31.9 ± 19.8 pg/mL at the first postoperative day (p = 0.0384) and rose again at the second postoperative day (52.1 ± 28.0 pg/mL) (Fig. 2).

**Table 1:** Patient characteristics and GIP serum levels

Parameter	Control (no myocardial infarction) (n=631)	STEMI (n=100)
GIP—pg/mL	271.54 (70.12–542.41)	216.82 (52.37–443.07)
Age—years	65.86 ± 12.53	62.24 ± 11.21
BMI—kg/m²	28.51 ± 5.75	26.17 ± 3.79
Male—No. (%)	475 (75.28)	84 (84)
Type 2 Diabetes—No. (%)	211 (33.44)	24 (24)
Hypertension—No. (%)	470 (74.84)	55 (55)
Smoker—No. (%)	123 (19.59)	47 (47)
Creatinine—mg/dL	1 (0.9–1.2)	0.9 (0.8–1.1)
eGFR—mL/min/1,73 m²	71.54 ± 22.89	81.76 ± 18.25
CK—U/L	87 (61–142)	197 (110–481)
Troponin—pg/mL	18 (10–38)	1428.5 (352.5–-2811)
CRP—mg/L	2.2 (2.2–9)	13 (7–33)
Leukocytes—nL	7.57 ± 2.32	9.22 ± 2.55
Coronary artery disease—No. (%)	457 (72.42)	100 (100)

Continuous variables are expressed as mean ± SD or median (Q1–Q3) in case of heavily skewed data. Categorical variables are shown as absolute (No.) and relative frequencies (%)

*BMI* body-mass-index, *CK* creatine kinase, *CRP* C-reactive protein, *eGFR* estimated glomerular filtration rate, *GIP* glucose-dependent insulinotropic polypeptide, *STEMI* ST-elevation myocardial infarction

**Table 2:** Correlation of baseline patient characteristics with GIP serum levels

Parameter	*ρ*	N	p-value
Age—years	0.0557	731	0.1324
BMI—kg/m²	0.0761	724	0.0407
Sex (male)	− 0.0163	731	0.6607
Type 2 Diabetes	0.0828	731	0.0252
Hypertension	0.0320	728	0.3885
Smoker	− 0.0579	728	0.1187
Creatinine—mg/dL	0.0497	638	0.2096
eGFR—mL/min/1,73 m²	− 0.0611	638	0.1234
CK—U/L	− 0.0387	610	0.3399
Troponin**—**pg/mL	− 0.0097	351	0.8562
CRP—mg/L	0.0924	607	0.0228
Leukocytes**—**nL	− 0.0455	683	0.2352
Coronary artery disease	− 0.0247	731	0.5052

*BMI* body-mass-index, *CK* creatine kinase, *CRP* C-reactive protein, *eGFR* estimated glomerular filtration rate, *ρ*  Spearman correlation coefficient, *N* number of observations, P-value of the test that Spearman’s rank correlation coefficient $\rho \ne 0$

## Discussion

Under physiological conditions GIP is secreted from enteroendocrine K-cells in the gut following food ingestion [25]. This study demonstrates that circulating GIP levels are reduced in patients after acute myocardial infarction or cardiac surgery. These findings might suggest nutrition-independent regulation of GIP secretion following acute myocardial injury in humans and warrant further investigations.

Nutrient-independent secretion of GLP-1 has been extensively studied in the past. We and others observed that inflammatory stimuli including LPS (lipopolysaccharide), IL-6 (interleukin 6) and IL-1b (interleukin 1b) directly induce GLP-1 secretion from intestinal L-cells through IL-6 signaling [26, 27]. Furthermore, gut intraepithelial lymphocytes were also identified to regulate systemic GLP-1 availability [28]. Consistently mice and patients with acute (sepsis [27] or myocardial infarction [29]) or chronic inflammatory cardiovascular diseases (coronary artery disease (CAD) [30] or heart failure [31]) show strongly increased circulating GLP-1 levels independent of food intake. Elevated GLP-1 levels were independently associated with mortality in patients with sepsis or myocardial infarction [32, 33]. Mechanistic experimental studies suggested upregulated GLP-1 secretion as an endogenous protective counter-regulatory response in terms of cardiovascular and inflammatory diseases [29].

In contrast to GLP-1, nutrition-independent regulation of GIP secretion is not well understood. In mice LPS and IL-1b administration directly induced GIP secretion and patients with peripheral arterial disease (PAD) showed higher circulating GIP levels compared to patients without PAD [21, 34]. However, in contrast to GLP-1, patients with CAD presented with similar circulating GIP levels compared to patients without CAD and GIP was not associated with inflammatory markers [21]. To the best of our knowledge this is the first study investigating endogenous GIP levels in humans following acute myocardial injury or inflammatory challenge. While GLP-1 has been identified and established over the last 10 years as a pleiotropic cardiovascular protective peptide beyond its glucoregulatory role, the relevance of GIP for CVD remains largely unknown. Interestingly, experimental studies found activation of the GIP receptor to exhibit various protective effects in cardiovascular murine disease models (reduction of atherosclerosis, stabilization of atherosclerotic plaques, suppression of cardiac hypertrophy and fibrosis in heart failure models), which does require translational investigations in humans [20–23, 35]. Recently we found circulating GIP levels in patients with acute myocardial infarction to be associated with favorable cardiovascular prognosis. In these patients higher GIP levels independently predicted reduced cardiovascular mortality [24]. Bioactive GIP and GLP-1 are rapidly inactivated by the enzyme dipeptidyl peptidase 4 (DPP-4). DPP-4 inhibitors improve glucose metabolism and are clinically used for the treatment of diabetes mellitus [3]. However, in several large CV outcome trials DPP-4 inhibitors proved CV safety, but failed to reduce CV endpoints and mortality [36–38]. Importantly, DPP-4 inhibitors are not limited to activate GIP and GLP-1 signaling. Next to GIP and GLP-1 DPP-4 has more than 60 other substrates including GLP-2, substance P, neuropeptide Y, stromal cell-derived factor-1α/β (CXCL12), GM-CSF (granulocyte macrophage colony-stimulating factor), CXCL10 and RANTES (Regulated and normal T-Cell-Expressed and Secreted) [39]. Thus, the question how activation of the GIP system directly affects CV prognosis in patients remains open. Due to lack of specificity DPP-4 inhibitors might not be the optimal tool to investigate CV outcome effects of GIP in patients. Future clinical trials with specific GIP receptor agonists are needed to foster our understanding of the gut-heart axis as a yet fairly neglected field of system biology and to elucidate whether the GIP system might open novel therapeutic approaches for the treatment of patients with CVD.

This study has several limitations. The underlying mechanism leading to the reduction of GIP after myocardial infarction or cardiac surgery remains currently unknown. Since GIP levels rose at the late time point after cardiac surgery (second post-operative day) without enteral or parenteral nutrition it appears unlikely that food restriction was the underlying mechanism of reduced GIP levels. Future mechanistic experimental and clinical studies investigating the interplay between GIP and other metabolic hormones are necessary to improve our understanding of GIP regulation in response to acute myocardial injury. We here report lower circulating GIP concentrations in response to myocardial injury. However, this observation does not imply a direct reduction in GIP secretion, since we only measured total GIP (including active and inactive cleaved peptides) and not active GIP (1-42) levels. Lower total GIP levels could also be related to changes in distribution and elimination. Finally, we have no information on food intake in Clinical Study I, which could have affected GIP secretion.

## Conclusions

Circulating GIP levels are downregulated in patients with myocardial infarction and following cardiac surgery. These results might suggest nutrition-independent regulation of GIP secretion following myocardial injury in humans. Upcoming studies are needed to elucidate whether GIP might be a novel therapeutic target in CVD.

## Data Availability

Data will be provided upon request to the corresponding author.

## Abbreviations

BMI: Body-mass-index
CAD: coronary artery disease
CRP: C-reactive protein
CVD: Cardiovascular disease
GLP-1: Glucagon-like peptide-1
GIP: Glucose-dependent insulinotropic peptide
GM-CSF: Granulocyte macrophage colony-stimulating factor
ICU: Intensive care unit
IL-1b: Interleukin 1b
IL-6: Interleukin 6
LPS: Lipopolysaccharide
PAD: Peripheral arterial disease
RANTES: Regulated and normal T-Cell-Expressed and Secreted
STEMI: ST-elevation myocardial infarction
WBC: White blood cells
